# Effects of auriculotherapy on anxiety and biomarkers in Primary Health Care: a clinical trial

**DOI:** 10.1590/0034-7167-2022-0728pt

**Published:** 2023-12-04

**Authors:** Carina da Silva, Letícia Siqueira, Lívia Crespo Drago, Elisa Mitkus Flores Lins, Daniel Fernandes Martins, Franciane Bobinski

**Affiliations:** I Universidade do Sul de Santa Catarina. Palhoça, Santa Catarina, Brazil

**Keywords:** Anxiety, Auriculotherapy, Biomarkers, Primary Health Care, Nursing Assessment, Ansiedade, Auriculoterapia, Biomarcadores, Atenção Primária à, Saúde, Avaliação em Enfermagem, Ansiedad, Auriculoterapia, Biomarcadores, Atención Primaria de Salud, Evaluación en Enfermería

## Abstract

**Objective:**

to assess the effects of auriculotherapy on anxiety and brain-derived neurotrophic factor (BDNF), neuron-specific enolase (NSE) and S100 calcium-binding protein B (S100B) serum levels in adults assisted in Primary Health Care.

**Methods:**

a pre-experimental pilot clinical trial. Information was obtained from 19 patients using the State-Trait Anxiety Inventory (STAI) and analysis of BDNF, NSE and S100B serum levels.

**Results:**

the pre-intervention anxiety score in the IDATE-Trait was 52.11±6.691 (CV 12.84%) and the assessment after auriculotherapy was significantly lower (43.72±8.141; CV 18.62%; P=0.0007). S100B levels were significantly reduced after auriculotherapy (from 64.03±72.18 to 54.03±68.53 pg/mL; CV 126.8%; P=0.0023).

**Conclusion:**

auriculotherapy effectively reduced anxiety levels. It proved to be safe and easy to apply, allowing nurses to perform this technique autonomously. A reduction of S100B was also evidenced, demonstrating possible prevention of neuronal damage.

## RESUMO

**Objetivo:**

avaliar os efeitos da auriculoterapia sobre a ansiedade e os níveis séricos do fator neurotrófico derivado do cérebro (BDNF), enolase-específica de neurônios (NSE) e proteína ligadora de cálcio S100B (S100B) em adultos atendidos na Atenção Primária à Saúde.

**Métodos:**

ensaio clínico piloto pré-experimental. As informações foram obtidas de 19 pacientes por meio do Inventário de Ansiedade Traço-Estado (IDATE) e da análise dos níveis séricos de BDNF, NSE e S100B.

**Resultados:**

o escore de ansiedade pré-intervenção no IDATE-Traço foi de 52,11±6,691 (CV 12,84%) e a avaliação após a auriculoterapia foi significativamente menor (43,72±8,141; CV 18,62%; P=0,0007). Os níveis de S100B foram significativamente reduzidos após auriculoterapia (de 64,03±72,18 para 54,03±68,53 pg/mL; CV 126,8%; P=0,0023).

**Conclusão:**

a auriculoterapia reduziu efetivamente os níveis de ansiedade. Mostrou-se segura e de fácil aplicação, possibilitando ao enfermeiro realizar esta técnica com autonomia. Uma redução de S100B também foi evidenciada, demonstrando possível prevenção de danos neuronais.

## RESUMEN

**Objetivo:**

evaluar los efectos de la auriculoterapia sobre la ansiedad y los niveles séricos de factor neurotrófico derivado de cerebro (BDNF), enolasa-específica de neurona (NSE) y proteína fijadora de calcio S100B (S100B) en adultos atendidos en Atención Primaria de Salud.

**Métodos:**

ensayo clínico piloto preexperimental. Se obtuvo información de 19 pacientes mediante el Inventario de Ansiedad Estado-Rasgo (STAI) y análisis de niveles séricos de BDNF, NSE y S100B.

**Resultados:**

la puntuación de ansiedad preintervención en el IDATE-Rasgo fue de 52,11±6,691 (CV 12,84%) y la valoración tras la auriculoterapia fue significativamente menor (43,72±8,141; CV 18,62%; P=0,0007). Los niveles de S100B se redujeron significativamente después de la auriculoterapia (de 64,03±72,18 a 54,03±68,53 pg/mL; CV 126,8%; P=0,0023).

**Conclusión:**

la auriculoterapia redujo efectivamente los niveles de ansiedad. Demostró ser seguro y fácil de aplicar, lo que permitió a las enfermeras realizar esta técnica de forma autónoma. También se evidenció una reducción de S100B, lo que demuestra una posible prevención del daño neuronal.

## INTRODUCTION

Mental health is currently a much discussed topic, as the proportion of people who have some type of mental disorder has grown exponentially around the world, pointing to several consequences in terms of loss of quality of life^(1)^. A 2017 World Health Organization (WHO) report estimated that approximately 264 million people in the world suffer from anxiety disorders, mainly affecting women between 40 and 49 years of age and appearing as the sixth cause of loss of quality of life for years lived with disability. In Brazil, the prevalence of anxiety disorders in the population reaches 9.3%, which is the highest rate among all those recorded in the report^(1)^.

Anxiety, as a nursing diagnosis, is defined as: “a vague and uncomfortable feeling of discomfort or fear, accompanied by an autonomic response, a feeling of apprehension, caused by the anticipation of danger”^(2)^. Aiming to overcome these altered emotional states, people seek Integrative and Complementary Practices (ICP) as a means of controlling and treating their illness, thus improving their quality of life^(3)^.

ICPs are therapeutic resources that aim to promote and restore health through the stimulation of natural mechanisms, using effective and safe technologies. Currently, there is a specific resource for implementing these practices in Brazil within the Brazilian Health System (SUS - *Sistema Único de Saúde*) called the Brazilian National Policy on Integrative and Complementary Practices (PNPIC - *Política Nacional de Práticas Integrativas e Complementares*), published as Ministerial Ordinance 971 on May 3, 2006 and later in a new Ordinance 1600, on July 17, 2006. The treatments offered, *a priori*, included Traditional Chinese Medicine (TCM)/acupuncture, homeopathy, phytotherapy, medicinal plants, social thermalism/crenotherapy, and anthroposophical medicine. In 2017 and 2018 the PNPIC was expanded and now covers 29 ICPs^(4-6)^. The Federal Council of Nursing (COFEN) determined and certified, through Resolution 326/2008, acupuncture as a specialty that can be performed by nurses, subject to proof of specific technical training and that must be applied complementarily in their activities, always aiming at the promotion and recovery of health and rehabilitation in illness^(7)^.

Auriculotherapy has its origins in TCM and includes techniques that use stimulation of specific points in the ear to treat physical and psychological illnesses^(8)^. This ancient technique considers the human organism as an energy field, causing the stimulation of the points to release *Qi*; i.e., in the Western view, it favors the release of neurotransmitters in the body, promoting treatment for various pathologies^(9)^. Anxiety treatment through auriculotherapy has been analyzed by several health professionals. A report with multiple cases carried out with nursing professionals scheduled to work during the Coronavirus pandemic proved through tests that auriculotherapy was effective in reducing emotional disorders such as anxiety^(10)^. Another study carried out with male patients undergoing coronary angiography proved that auriculotherapy reduced patients’ anxiety before the procedure^(11)^. An integrative literature review carried out in 2014 revealed that 78.11% of the analyzed studies indicated that auriculotherapy is an effective intervention to reduce anxiety levels, including reflecting on physiological data such as heart rate and blood pressure^(8)^.

Several lines of evidence point to the need to explore the relationship between anxiety and peripheral brain-derived neurotrophic factor (BDNF) levels. BDNF is a protein widely distributed by the central nervous system (CNS) that has a neurotrophic effect on the development and function of neurons, in particular on serotoninergic neurotransmission, having antidepressant and anxiolytic effects^(12)^. Studies have reported inverse correlations between BDNF and anxiety-related symptoms^(13-14)^. However, the relationship of BDNF levels in subjects with anxiety symptoms treated with auriculotherapy remains unknown.

Studies have also investigated the importance of other anxiety-related biomarkers, such as neuron-specific enolase (NSE) and S100 calcium-binding protein B (S100B). NSE is a glycolytic enzyme found mainly in neurons and neuroendocrine cells, which can perform neuroinflammation, neurodegeneration, and neuroprotection functions^(15)^. Research shows that the extracellular presence of NSE is indicative of neuronal damage in pathological situations, in addition to pointing to the emergence of alterations in serum levels of this enzyme in individuals with mood disorders^(16)^. S100B is a calcium-binding protein present mainly in the cytoplasm of astrocytes, and its levels in biological fluids also indicate neural damage^(17)^. According to controlled scientific experiments, elevated S100B serum levels are directly related to significantly higher states of anxiety as well as impaired cognitive functions^(18-19)^.

With the increase in anxiety in the population, there is a growing search for treatments that alleviate symptoms and bring better quality of life for these people. A survey carried out in a psychology service-school from 2009 to 2014 showed that anxiety together with depressive symptoms, among other complaints, were the main reasons for seeking care^(20)^. Knowledge of these data reinforces the importance of using auriculotherapy as a nursing intervention, highlighting the relevance of nursing consultations and diagnoses, as nurses are professionals who can carry out specializations to apply this therapy according to the COFEN Resolution 585/2018^(21)^. According to a study carried out with Primary Health Care (PHC) nurses, a nursing consultation is an extremely important means for developing clinical practice, anchored on the principle of comprehensiveness and evidence-based practice, contributing to improving the quality of care provided and recognizing nurses’ autonomy^(22)^. Reinforcing this idea, a survey carried out with workers at a Basic Health Unit (BHU) evidenced professionals’ willingness to implement ICPs in PHC for treating mental disorders. Thus, 73.9% claim to know an IPC (of which 92.9% of professionals mention TCM/acupuncture as a known practice); 94.2% acknowledge that BHU users with mental health issues would benefit from the therapies offered; 91.3% claim they would like to receive training in ICPs; and 92.8% report that they consider ICPs as a way to perform mental health care^(23)^.

The present study aims to apply auriculotherapy as a tool in anxiety control and treatment, being a safe, low-cost, non-invasive, and quick-to-perform technique, which can be applied autonomously by nurses. It is necessary to highlight the evidence of its effect on improving quality of life as well as greater knowledge about the technique among PHC professionals, in order to favor the implementation of the procedure and promote the prevention and early treatment of anxiety and related harms.

## OBJECTIVE

To assess the effects of auriculotherapy on anxiety and BDNF, NSE, and S100B serum levels in adults in PHC in a BHU in the city of Palhoça, Santa Catarina.

## METHODS

### Ethical aspects

The study was conducted in accordance with national and international ethics guidelines, approved by the Research Ethics Committee of the *Universidade do Sul de Santa Catarina* and Certificate of Presentation for Ethical Consideration (CAAE - *Certificado de Apresentação para Apreciação Ética*), whose opinion is attached to this submission. This clinical trial was published in the Brazilian Registry of Clinical Trials (ReBEC) under RBR-35n9nkj. The written Informed Consent Form was obtained from all individuals involved in the study.

### Design, period and place

**Figure 1:**
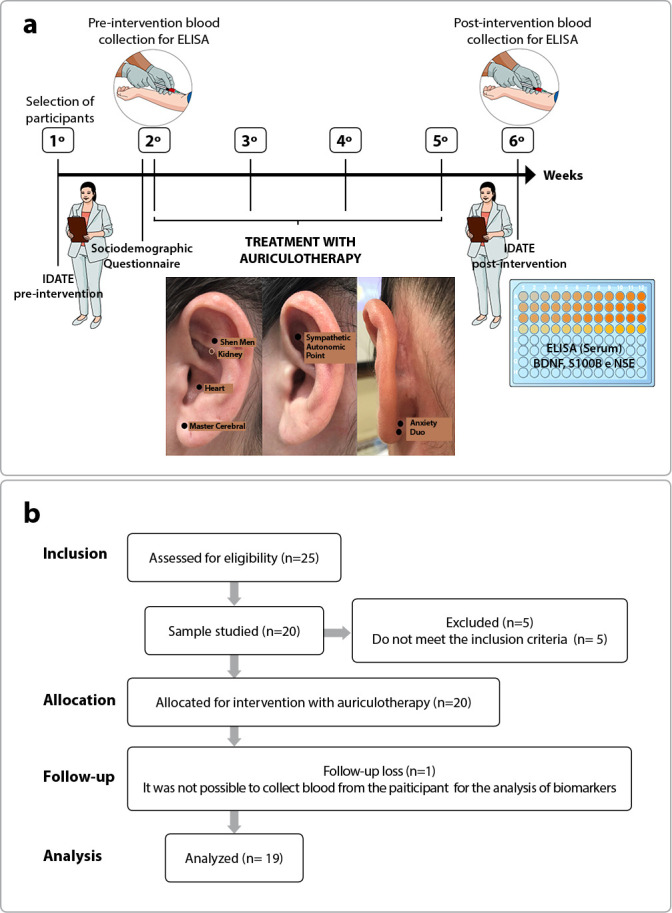
Timeline of procedures performed in the study (a) and CONSORT flowchart (b)

This study is characterized as a pre-experimental pilot clinical trial with a quantitative approach, carried out at a BHU in the city of Palhoça, Santa Catarina, in which the period of data collection was from July to September 2019. The study was carried out over six weeks: in the first meeting, the ICF was read and signed, and STAI was applied to assess anxiety levels and eligibility in the sample; in the second meeting, a sociodemographic questionnaire was applied, blood was collected for analysis of serum BDNF, NSE, and S100B, and treatment with auriculotherapy was started; in the third, fourth, and fifth meetings, auriculotherapy sessions were repeated; in the sixth meeting, STAI was applied again to reassess anxiety levels and blood was collected for post-intervention BDNF, NSE, and S100B analysis (Figure 1a). The research was conducted and structured following the Consolidated Standards of Reporting Trials (CONSORT) guidelines.

*Caption: STAI - State-Trait Anxiety Inventory; ELISA - enzyme-linked immunosorbent assay; BDNF - brain-derived neurotrophic factor; S100B - S100 calcium binding protein B; NSE - neuron-specific enolase.*

### Sample and selection criteria

For sample selection, the survey was disclosed at the BHU among users and 25 participants voluntarily agreed to be part of the study. Participants over 18 years, of both sexes, with medium (from 41 to 60 points) and high (from 61 to 80 points) anxiety levels, measured using the State-Trait Anxiety Inventory (STAI), not being on psychotropic drugs, antibiotics, corticosteroids, or anabolic steroids, not being in the first trimester of pregnancy, and not being illiterate were included.

In order to characterize the sample, a sociodemographic questionnaire was applied, including the following variables: age, sex, education level, religion, family income, alcohol consumption, consumption of caffeinated beverages, use of illicit drugs, physical activity, and use of natural substances such as plants and phytotherapy.

### Anxiety assessment

The STAI, developed by Spielberger^(24)^, translated and validated to Portuguese by Biaggio and Natalício^(25)^, was used to assess anxiety before and after the intervention with auriculotherapy. It is an instrument composed of two self-assessment subscales: the STAI-Trait (STAI-T), which defines an individual’s anxiety trait, pointing out the tendency to react to situations identified as threatening, with the objective of becoming more stable, proposing that individuals describe how they generally feel. The second subscale is the STAI-State (STAI-S), which identifies the state of anxiety in relation to a specific situation considered to be worrying or distressing and intends to be a transitory characteristic, asking individuals to describe how they feel at the time they respond to the questionnaire. Each of the subscales has 20 questions, with four possible degrees of response intensity, ranging from 1 to 4, so that the scores for each volunteer range from 20 to 80 points, which may indicate low (20 to 40), medium (41 to 60), and high (61 to 80) anxiety levels.

### Auriculotherapy treatment

The technique consists of stimuli to specific points in the ear, which correspond to each region of the body as microsystems, with neurophysiological bases. As the ear is considered a reflex area, when stimulated, it connects via afferent nerve pathways to the CNS, and from there to the autonomous nervous system (ANS). This stimulus provokes responses that help in therapeutic treatment, forming the triple relationship: auricular point, brain, and organs^(26)^. In the auriculotherapy intervention, the following materials were used: mustard seeds (arranged on a card covered with an anti-allergic adhesive); tweezers suitable for applying the seeds; cotton, and 70% ethyl alcohol. The procedure was performed after inspection and choice of the dominant auricle of each participant, applying the seeds in a set of points in the following order of application: *Shenmen*; Kidney; Neurovegetative System; Heart; Joy; and Anxiety II^(27)^ (Figure 1). After the appropriate location of the points, the ear pinna was cleaned with cotton and 70% alcohol, and the application of the seeds was affixed with adhesive tape. The therapy lasted for four weeks, with a total of 4 applications of auriculotherapy per patient (alternating the ear), performed once a week.

### Blood collection and biochemical analysis

Blood collection was performed before and after the auriculotherapy intervention. Blood samples were placed in vacutainer tubes (BD Vacutainer^*®*^ SST^*®*^ II, BD) without anticoagulant, and left to stand at room temperature for 20 minutes. Next, they were centrifuged at 3,000 rpm for 10 minutes and stored at a temperature of 4º C for a maximum of 4 hours. Subsequently, aliquots of the supernatant (serum) were stored at -80°C until the time of assay.

Analyses were performed by enzyme-linked immunosorbent assay (ELISA). For the assay, 100 µl of sample was used to measure the concentrations of BDNF, S100B, and NSE (DY248, DY1820, DY5169, DuoSet ELISA Human, R&D Systems, Minneapolis, MN, USA), according to the kit manufacturer’s instructions. The values obtained were estimated by interpolating the data with a standard curve for each biomarker using a colorimetric assay, measured at 450 nm (wavelength correction 540 nm) in a spectrophotometer (Perlong DNM-9602, Perlong Medical Equipment Co., Nanjing, China), and the results were expressed in picograms per milliliter (pg/mL).

### Data analysis

The information obtained through STAI, sociodemographic questionnaire, and serum concentrations of BDNF, NSE, and S100B were organized in Excel^®^ spreadsheets and analyzed using the Graph Pad Prism^®^ program (v. 8.0, La Jolla, California, USA). Initially, the normal data distribution was assessed by the Shapiro-Wilk test and, therefore, the results were presented as mean ± standard deviation (SD) and coefficient of variation (CV). Parametric data from biochemical and STAI assessments before and after the auriculotherapy intervention were compared using the paired Student’s t test. To analyze the correlation between BDNF, NSE, and S100B serum levels and anxiety levels, the Pearson correlation test was used, in addition to the confidence interval (CI) and the coefficient of determination (R^2^). The correlation strength will be interpreted within the range of -1 to +1. Sociodemographic variables were presented as absolute numbers and percentages. Multiple linear regression was performed using the backward stepwise method. The validity of final multiple regression models required the following assumptions: (a) no heteroskedasticity (Breusch-Pagan hettest); (b) no multicollinearity (variance inflation factor); (c) residuals normality distributed (Shapiro-Wilk normality test). Multiple linear regression was performed on Stata 14.0 (Version 14; StataCorp LLC, Texas, 200 USA) statistical package. In all analyses, p-values less than 0.05 were considered statistically significant.

## RESULTS

Initially, 25 participants were recruited. After applying the eligibility criteria, 20 participants met the inclusion criteria and were allocated to the intervention, however there was a loss of 1 participant following the research, thus 19 participants were analyzed (Figure 1b).

With respect to sociodemographic data, 4 participants were between 18 and 25 years of age (21.05%); 10 were between 26 and 35 years (52.63%); 2 were between 36 and 45 years (10.53%); and 3 were over 45 years (15.79%). Moreover, 7 were male (36.85%) *versus* 12 were female (63.15%). As for education, 1 had from 6 to 9 years of study (5.26%); 2 had from 9 to 12 years of study (10.53%); 9 had from 12 to 15 years of study (47.36% ); 7 had more than 15 years of study (36.85%). Also, 11 had a religion (57.89%); 8 had no religion (42.11%); 13 did not use illicit drugs (68.43%); 5 used them once a week (26.31%); 1 used them five times a week (5.26%); 10 did not practice physical activity regularly (minimum 30min per day) (52.63%); 4 practiced twice a week (21.06%); 3 practiced three times a week (15.79%); 1 practiced four times a week (5.26%); and 1 practiced five times a week (5.26%). Other sociodemographic characteristics of the sample are shown in Table 1.

**Table 1:** Sociodemographic data

Variable	n	%
Age		
18 - 25 years	4	21.05
26 - 35 years	10	52.63
36 - 45 years	2	10.53
> 45 years	3	15.79
Sex		
Male	7	36.85
Female	12	63.15
Education level		
From 6 to 9 years of study	1	5.26
From 9 to 12 years of study	2	10.53
From 12 to 15 years of study	9	47.36
More than 15 years of study	7	36.85
Religion		
Yes	11	57.89
No	8	42.11
Family income		
Up to R$ 1,000.00	1	5.26
From R$ 1,000.00 to 2,000.00	1	5.26
From R$ 2,000.00 to 3,000.00	7	36.85
From R$ 3,000.00 to 4,000.00	2	10.53
From R$ 4,000.00 to 5,000.00	2	10.53
More than R$ 5,000.00	6	31.57
Alcohol consumption		
Do not consume	10	52.63
Consume 1x a week	5	26.32
Consume 2x a week	2	10.53
Consume 3x a week	1	5.26
Consume 7x a week	1	5.26
Consumption of caffeinated beverages		
Do not consume	3	5.79
Consume 1x a week	1	0.26
Consume 2x a week	1	0.26
Consume 3x a week	1	0.26
Consume 5x a week	1	0.26
Consume 6x a week	2	0.53
Consume 7x a week	10	2.64
Use of illicit drugs		
Do not use	13	8.43
Use 1x a week	5	6.31
Use 5x a week	1	0.26
Physical activity practice		
Do not practice	10	5.63
Practice 2x a week	4	1.06
Practice 3x a week	3	5.79
Practice 4x a week	1	0.26
Practice 5x a week	1	0.26
Use of natural substance (plants/phytotherapy)		
Do not use	7	6.84
Use 1x a week	2	0.53
Use 3x a week	2	0.53
Use 5x a week	1	0.26
Use 7x a week	7	6.84

*Caption: bold used to highlight the title of the questions of the sociodemographic characteristics of study participants. N - number of sample participants.*

**Figure 2:**
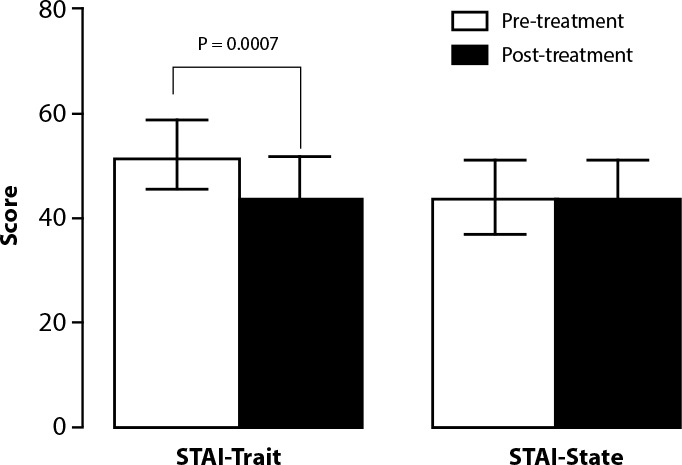
Pre-treatment and post-treatment assessment after auriculotherapy on STAI-Trait and STAI-State

*Note: data are expressed as mean ± SD compared statistically using paired Student’s t test.*

Figure 2 shows the total STAI-T and STAI-S scores. The mean pre-intervention anxiety score on the STAI-T was 52.11 ± 6,691 (CV 12.84%), and the post-intervention assessment after auriculotherapy showed significantly lower rates (43.72 ± 8,141; CV 18.62%; P = 0.0007). The STAI-S presented a pre-intervention anxiety score of 44.05 ± 6,998 (CV 15.86%) and post-intervention of 44.32 ± 7.095 (CV 16.01%); this difference was not statistically significant (P > 0.05) (Figure 2).

**Figure 3:**
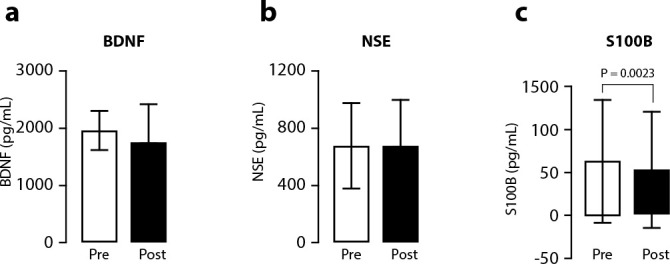
Effect of auriculotherapy treatment on biochemical markers (panel a BDNF; panel b NSE; panel c S100B)

*Note: data are expressed as mean ± SD compared statistically using paired Student’s t test.*

Neuronal biomarker analyses were compared using pre-treatment versus post-treatment, as shown in Figure 3. Pre-treatment BDNF levels (1964 ± 345.1 pg/mL, CV 17.58%) were not significantly changed when compared to post-treatment levels (1729 ± 707.9 pg/ml, CV 40.94%, P > 0.05, Fig. 3a). NSE concentration also showed no statistically significant difference between pre-treatment (676.5 ± 304.1pg/mL; CV 44.95%) versus post-treatment values (668 ± 332.6 pg/mL; CV 49.80%; P > 0.05 Fig. 3b). Surprisingly, S100B levels that were at 64.03 ± 72.18 pg/mL pre-intervention were significantly reduced after treatment with auriculotherapy (54.03 ± 68.53 pg/mL; CV 126.8%; P = 0.0023 Fig. 3c).

**Figure 4:**
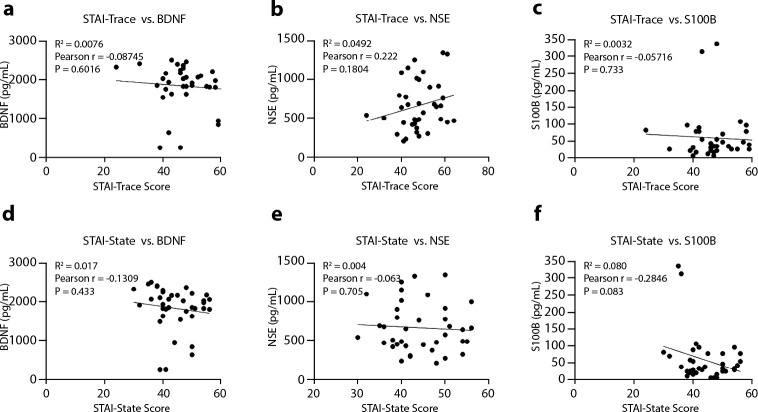
Correlations of biochemical markers with STAI-Trait and STAI-State

*Note: correlation panel a STAI-Trait versus BDNF; correlation panel b STAI-Trait versus NSE; correlation panel c STAI- Trait versus S100B; correlation panel d STAI-State versus BDNF; correlation panel e STAI-State versus NSE; correlation panel f STAI-State versus S100B.*

BDNF, NSE, and S100B serum levels were correlated with STAI-Trait and STAI-State scores, using the Pearson correlation test (Figure 4). The STAI-Trait versus BDNF score showed a weak correlation (Pearson’s r = -0.08745; P = 0.6016; CI from -0.3961 to 0.2389; R^2^=0.0076; Fig. 4a). The STAI-State versus BDNF demonstrated a weak correlation (Pearson’s r = -0.1309; P=0.433; CI from -0.4325 to 0.1970; R^2^ = 0.017; Fig. 4d). The STAI-Trait versus NSE demonstrated a weak correlation (Pearson’s r = 0.222; P = 0.1804; CI from -0.1051 to 0.5058; R^2^ = 0.0492; Fig. 4b). The STAI-State versus NSE demonstrated a weak correlation (Pearson’s r = -0.063; P = 0.705; CI from -0.3754 to 0.2617; R^2^ = 0.004; Fig. 4e). The STAI-Trait versus S100B demonstrated a weak correlation (Pearson’s r = -0.05716; P = 0.733; CI from -0.3701 to 0.2674; R^2^ = 0.0032; Fig. 4c). The STAI-State versus S100B demonstrated a weak correlation (Pearson’s r = -0.2846; P = 0.083; CI from -0.5539 to 0.03863; R^2^ = 0.080; Fig. 4f). Table 2 shows multiple linear regression model for the association between clinical variables (STAI-Trait and STAI-State and biochemical markers) with sociodemographic variables suggests that physical activity (P = 0.014) and age (P = 0.019) are significantly associated with 45% of change in BDNF difference after treatment (R^2^ = 0.45, P = 0.007). Thus, the final regression model suggests that sex (P = 0.022), family income (P = 0.004) and use of natural substances (P = 0.023) are significantly associated with 58% of change in STAI-Trait difference after treatment (R^2^ = 0.58, P = 0.0035). STAI-State and NSE and S100B biochemical markers are not associated with sociodemographic variables in the final regression model (Table 2).

**Table 2:** Final multiple linear regression model

Variable	R^2^ coefficient	Adjusted R^2^	B coefficient (95%CI)	*p* level
BDNF	0.45	0.38		0.007
Regular physical exercise, yes or no			849.12 (198.34 - 1499.90)	0.014
Age, years			-31.02 (-56.23 - -5.80)	0.019
Constant			898.70 ( -56.97 - 1854.38)	0.064
STAI-Trait	0.58	0.50		0.035
Sex, female or male			7.76 (1.308068 - 14.2169)	0.022
Family income			-3.19 (-5.1899 - -1.1964)	0.004
Use of natural substances, yes or no			- 7.58 (-13.991 - -1.1768)	0.023
Constant			21.36 (11.359 - 31.370)	0.000

*Final regression model suggesting that BDNF is positively associated with regular physical activity, and inversely associated with age. IDATE-Trait is associated with female sex, inversely associated with income and use of natural substances.*

## DISCUSSION

According to high anxiety levels in Brazil and the suffering experienced by people with this disorder, there is a search for alternative treatments to alleviate the relevant symptoms of this disease. This emphasizes the importance of investigating auriculotherapy as a technique that meets the demand of these individuals. In the current study, it was found that the manifestation of anxiety disorder was reversed or controlled, indicating that the observed results can be considered positive and beneficial. It was also found that after four weeks of treatment with auriculotherapy, 89.42% of patients already demonstrated a reduction in anxiety levels, confirming previous studies. A field study with a qualitative approach, using the Hamilton Anxiety Rating Scale, with ear points Kidney, *Shenmen*, Heart, and Anxiety, for eight weeks, showed that auriculotherapy reduced participants’ anxiety levels by 71.43%^(28)^. In Cuba, an experimental study on a therapeutic intervention carried out with 100 patients with anxiety disorder, divided into a study group and a control group, using the points *Shenmen*, Heart, Kidney, and Zero Point, showed that 92% of patients reported remission of almost all anxiety symptoms after four weeks of treatment^(29)^.

It was found that before treatment 84.17% of patients presented a medium anxiety score and 15.83% a high anxiety score, while the majority had reduced scores after treatment demonstrated using the STAI-T. In the STAI-S, 100% of participants presented medium anxiety, showing no significant decrease in scores after treatment. According to the results presented, it can be seen that there is a difference between Trait and State scales scores. The significant result was demonstrated through the STAI-T, which refers to a relatively stable personal disposition and mentions the tendency to react to situations understood as threatening, presenting itself as a personality trait. This finding agrees with previous studies that demonstrate that the anxiety trait is related to individual characteristics, and scores are less susceptible to variations arising from the environmental context, maintaining a certain constancy over time^(30)^. Furthermore, the final regression model showed an association between IDATE-T and females, lower income and in those who did not use natural substances. Our data corroborate the literature that highlights that women are more affected by anxiety disorders than men, and this difference became more evident globally during the COVID-19 pandemic^(31-32)^, period in which correlates for poorer mental health also included people with lower income^(32)^. Conventional management of anxiety disorders is based on pharmacotherapy and psychotherapy; however, many patients prefer natural products for symptom relief to conventional medications. The literature shows that herbal supplementation is an effective method for treating anxiety and anxiety-related conditions without the risk of serious side effects, and our study confirms that patients who not used natural products had a higher T-STAI score^(33)^. The STAI-S, on the other hand, did not demonstrate a relevant statistical difference in this study, as it is a transitory condition where individuals may have gone through momentary situations of distress, unease, and anguish, reflecting in this score. This corroborates previous studies that relate the anxiety state score to the momentary emotional condition, which may vary according to environmental situations and oscillate in time^(30)^.

Several lines of research highlight the need to explore the relationship between BDNF and anxiety peripheral levels as well as the importance of other biomarkers such as NSE and S100B. Studies show that BDNF is involved in neuroplasticity of the nervous system, including neuronal maturation and synaptic remodeling, suggesting that it may be involved in anxiety’s neurobiology, representing a useful biomarker for this type of investigation^(34)^. However, in the current study, there was no statistically significant alteration in serum BDNF levels pre versus post-treatment with auriculotherapy as well as no correlation between anxiety scores and serum BDNF levels. Our results corroborate previous findings, where no difference was found in BDNF levels between anxiety patients and controls, regardless of the type of anxiety disorder presented^(35)^.

When we correlated biochemical markers with sociodemographic variables, the final regression model showed that BDNF is positively associated with regular practice of physical activity, and inversely associated with age. Our results corroborate a large body of evidence in the literature, which shows that BDNF peripheral levels increase with physical exercise in healthy people and in those with comorbidities such as depression, cognitive impairment, anxiety and neurodegenerative diseases. The literature suggests that the increase in BDNF levels and its tropomyosin receptor kinase B (TrkB) after physical exercise causes morphological and functional changes in the spinal cord, substantia nigra and in the neurons of the hypothalamic nuclei, responsible for: the alteration of the functional properties of the motoneurons that innervate the skeletal muscles; the increase in the release of dopamine in the brain; and the modulation of hormone levels involved in the regulation of metabolic processes supporting the benefits of exercise on brain health for its neuroprotective effect^(36)^. It is not fully defined whether BDNF levels decrease with age in healthy people, however this reduction is well established in elderly patients with neuropsychiatric and neurodegenerative diseases. Probably, the association of reduced BDNF levels with age in this study is also linked to the anxiety condition of these participants^(37-38)^.

Studies indicate that NSE is an enzyme associated with neuronal damage in pathological situations, in addition to pointing to the emergence of alterations in serum levels of this enzyme in individuals with mood disorders^(16)^. In the present study, no statistically significant alteration was found in serum levels of NSE preversus post-intervention with auriculotherapy as well as no correlation of anxiety levels with this biomarker. This finding is in line with research in which NSE levels and the severity of anxiety symptoms were not associated^(16)^.

S100B is a calcium-binding protein produced and secreted mainly by astrocytes, demonstrating that increased serum levels are indicative of neuronal injury or death^(17)^. In the current study, it was pointed out that S100B serum levels significantly reduced preversus post-intervention with auriculotherapy, suggesting a possible benefit of treatment. When we correlated S100B serum levels with anxiety, there was no statistically significant evidence. Although high S100B levels are related to schizophrenic patients^(39)^ as well as patients with major depression^(40)^, to date, the correlation between S100B levels and anxiety has not been found in the literature, and it is not possible to compare our results with previous studies.

### Study limitations

As for the limitations of this study, the number of participants may have been insufficient to demonstrate better results from auriculotherapy treatment with seeds as well as to show significant correlations between anxiety levels and biomarkers. It is suggested that future studies do not exclude symptomatic patients undergoing drug treatment, as many symptoms of high anxiety cause great suffering in these individuals, and auriculotherapy should be integrated with first-choice drug treatments. Thus, the inclusion of these patients could have increased our sample size. Further studies using other materials are recommended, such as crystal spheres, gold, silver, magnets, or even needles as well as longer treatment times, which could lead to an even greater reduction in anxiety levels. In the current study, a standardized treatment protocol was used for all participants, which may have limited the results in reducing anxiety levels. It is possible an individualized protocol, considering the specific demands of each participant, could have presented better results. It is also suggested that other studies be carried out in a randomized manner, using a control group for better observations of treatment effects. Furthermore, it was not possible in this study to better control the data of participants who used illicit drugs and who consumed alcoholic beverages.

### Contributions in nursing

Auriculotherapy was shown to be an effective intervention in reducing anxiety levels as well as related signs and symptoms, proving to be a safe, non-invasive, easy-to-apply practice, with minimal side effects and low cost, enabling nurses to perform this technique with autonomy in PHC. A reduction in S100B serum levels was also evidenced, demonstrating possible prevention of neuronal damage.

## CONCLUSIONS

The importance of nursing consultations fostered in evidence-based practice is reinforced, observing people as a whole, meeting all their needs. It is considered essential that PHC provide ICPs, such as auriculotherapy, which is a practice that can be used as an adjunct in first-line treatment, aiming at health promotion and the prevention, control, and treatment of anxiety, among other illnesses.

## Supplementary Material

0034-7167-reben-76-06-e20220728-suppl01

0034-7167-reben-76-06-e20220728-suppl02

0034-7167-reben-76-06-e20220728-suppl03

## Data Availability

https://doi.org/10.48331/scielodata.EWMJPB
