# Supplementary material for: Effects of auriculotherapy on anxiety and biomarkers in Primary Health Care: a clinical trial
Source: Rev Bras Enferm. 2023 Dec 4;76(6):e20220728. doi: 10.1590/0034-7167-2022-0728pt (PMC10695056; doi:10.1590/0034-7167-2022-0728pt)
Supplement: 0034-7167-reben-76-06-e20220728-suppl01 [file 0034-7167-reben-76-06-e20220728-suppl01.pdf]

Multiple Linear Regression

DIFERENÇA BDNF
BDNF DIFFERENCE

```
. stepwise, pr(.05): regress BDNF Difference sex0male1female Age Level of education Family income
> r smoking0No1Yes Alcohol consumption 0No1Yes Consumption of caffeinated beverages 0No1Yes Practice of Physical Activity
> Use of Natural Substances   Trait-Pre State-Pre
                  begin with full model
p = 0.9122 >= 0.0500  removing Consumption of caffeinated beverages
p = 0.8830 >= 0.0500  removing Level of education
p = 0.8454 >= 0.0500  removing Use of Natural Substances
p = 0.7999 >= 0.0500  removing sex0male1female
p = 0.7481 >= 0.0500  removing Alcohol consumption 0No1Yes
p = 0.6617 >= 0.0500  removing smoking0No1Yes
p = 0.6226 >= 0.0500  removing State-Pre
p = 0.1512 >= 0.0500  removing Trait-Pre
p = 0.1003 >= 0.0500  removing Family income

      Source |       SS       df       MS              Number of obs =      19
-------------+------------------------------           F(  2,    16) =    6.75
       Model |  5994013.7     2  2997006.85            Prob > F      =  0.0075
    Residual |  7106372.25    16  444148.266           R-squared     =  0.4575
-------------+------------------------------           Adj R-squared =  0.3897
       Total |  13100385.9    18  727799.219           Root MSE      =  666.44

------------------------------------------------------------------------------------------
               BDNF Difference |      Coef.   Std. Err.      t    P>|t|     [95% Conf. Interval]
--------------------------+---------------------------------------------------------------
Practice of Physical Activity |   849.1216   306.9858    2.77   0.014     198.3408    1499.902
                          Age |  -31.02488   11.89605   -2.61   0.019    -56.24339   -5.806374
                        _cons |   898.7045   450.8118    1.99   0.064    -56.97376    1854.383
------------------------------------------------------------------------------------------
```

DIFERENÇA S100B
S100B DIFFERENCE

```
. stepwise, pr(.05): regress difference S100B sex0male1female Age Level of education Family
income
> r smoking0No1Yes Alcohol consumption 0No1Yes Consumption of caffeinated beverages
0No1Yes Practice of Physical Activity
> Use of Natural Substances   Trait-Pre State-Pre
                 begin with full model
p = 0.9425 >= 0.0500  removing smoking0No1Yes
p = 0.6180 >= 0.0500  removing sex0male1female
p = 0.4334 >= 0.0500  removing Level of education
p = 0.2082 >= 0.0500  removing Consumption of caffeinated beverages 0No1Yes
p = 0.1299 >= 0.0500  removing Practice of Physical Activity
p = 0.2576 >= 0.0500  removing Trait-Pre
p = 0.2290 >= 0.0500  removing Alcohol consumption 0No1Yes
p = 0.0875 >= 0.0500  removing Family incomer
p = 0.1014 >= 0.0500  removing Age
p = 0.3384 >= 0.0500  removing State-Pre
p = 0.1832 >= 0.0500  removing Use of Natural Substances

     Source |       SS       df       MS              Number of obs =      19
-------------+------------------------------           F(  0,    18) =    0.00
      Model |          0     0          .           Prob > F      =       .
   Residual |  2719.48114    18  151.082285           R-squared     =  0.0000
-------------+------------------------------           Adj R-squared =  0.0000
      Total |  2719.48114    18  151.082285           Root MSE      =  12.292

------------------------------------------------------------------------------
    difference S100B |      Coef.   Std. Err.      t    P>|t|     [95% Conf. Interval]
-------------+----------------------------------------------------------------
      _cons |   9.999022   2.819876     3.55   0.002     4.074682    15.92336
------------------------------------------------------------------------------
```

DIFERENÇA NSE
NSE DIFFERENCE

```
. stepwise, pr(.05): regress NSE difference sex0male1female Age Level of education Family incomer
>  smoking0No1Yes Alcohol consumption 0No1Yes Consumption of caffeinated beverages 0No1Yes Practice of Physical Activity
> Use of Natural Substances   Trait-Pre State-Pre
                  begin with full model
p = 0.9701 >= 0.0500  removing Family incomer
p = 0.8281 >= 0.0500  removing Practice of Physical ActivityPhysical Activity Frequency
p = 0.4473 >= 0.0500  removing Alcohol consumption 0No1Yes
p = 0.3434 >= 0.0500  removing smoking0No1Yes
p = 0.1633 >= 0.0500  removing Trait-Pre
p = 0.1110 >= 0.0500  removing Level of education
p = 0.0533 >= 0.0500  removing Consumption of caffeinated beverages 0No1Yes
p = 0.0789 >= 0.0500  removing State-Pre
p = 0.1963 >= 0.0500  removing Age
p = 0.1831 >= 0.0500  removing Use of Natural Substances
p = 0.1341 >= 0.0500  removing sex0male1female

     Source |       SS       df       MS            Number of obs =      19
-------------+------------------------------           F(  0,    18) =    0.00
      Model |          0     0          .          Prob > F      =      .
   Residual |  1423281.45    18  79071.1918          R-squared     =  0.0000
-------------+------------------------------           Adj R-squared =  0.0000
      Total |  1423281.45    18  79071.1918          Root MSE      =   281.2

------------------------------------------------------------------------------
     Difference NSE |      Coef.   Std. Err.      t    P>|t|     [95% Conf. Interval]
-------------+----------------------------------------------------------------
      _cons |   8.532737   64.51079     0.13   0.896    -126.9994    144.0649
------------------------------------------------------------------------------
```

DIFERENÇA ESCALA IDATE – TRAÇO
STAI SCALE DIFFERENCE - TRAIT

```
. stepwise, pr(.05): Regress Trait Difference sex0male1female Age Level of education Family
income
> r smoking0No1Yes Alcohol consumption 0No1Yes Consumption of caffeinated beverages
0No1Yes Practice of Physical Activity
> Use of Natural Substances  BDNF Difference S100B Difference NSE Difference
                begin with full model
p = 0.9988 >= 0.0500  removing Level of education
p = 0.8410 >= 0.0500  removing NSE Difference
p = 0.7727 >= 0.0500  removing smoking0No1Yes
p = 0.6800 >= 0.0500  removing Practice of Physical Activity
p = 0.5859 >= 0.0500  removing Alcohol consumption 0No1Yes
p = 0.4410 >= 0.0500  removing BDNF Difference
p = 0.0554 >= 0.0500  removing S100B Difference
p = 0.1235 >= 0.0500  removing Consumption of caffeinated beverages 0No1Yes
p = 0.0719 >= 0.0500  removing Age

     Source |       SS       df       MS              Number of obs =      19
-------------+------------------------------           F(  3,    15) =    7.07
      Model |  839.157485     3  279.719162           Prob > F      =  0.0035
   Residual |  593.263567    15  39.5509045           R-squared     =  0.5858
-------------+------------------------------           Adj R-squared =  0.5030
      Total |  1432.42105    18  79.5789474           Root MSE      =   6.289

------------------------------------------------------------------------------------------
               Trait Difference |      Coef.   Std. Err.      t    P>|t|     [95% Conf. Interval]
--------------------------+----------------------------------------------------------------
   sex0male1female |   7.762482    3.02818     2.56   0.022     1.308068     14.2169
          Family income |  -3.193198   .9368228    -3.41   0.004    -5.189989   -1.196408
Use of Natural Substances |  -7.584117   3.006048    -2.52   0.023    -13.99136   -1.176878
                 _cons |   21.36469   4.694286     4.55   0.000     11.35905    31.37032
------------------------------------------------------------------------------------------
```

DIFERENÇA ESCALA IDATE –ESTADO
IDATE - STATE SCALE DIFFERENCE

```
. stepwise, pr(.05): regress State difference sex0male1female Age Level of education Family income
> smoking0No1Yes Alcohol consumption 0No1Yes Consumption of caffeinated beverages 0No1Yes Practice of Physical Activity
> Use of Natural Substances  BDNF Difference S100B Difference NSE Difference
                  begin with full model
p = 0.9525 >= 0.0500  removing BDNF Difference
p = 0.8174 >= 0.0500  removing Use of Natural Substances
p = 0.6228 >= 0.0500  removing Consumption of caffeinated beverages 0No1Yes
p = 0.5288 >= 0.0500  removing S100B Difference
p = 0.3341 >= 0.0500  removing Family incomer
p = 0.1544 >= 0.0500  removing Alcohol consumption 0No1Yes
p = 0.2185 >= 0.0500  removing smoking0No1Yes
p = 0.1608 >= 0.0500  removing Age
p = 0.2281 >= 0.0500  removing NSE Difference
p = 0.3368 >= 0.0500  removing Practice of Physical Activity
p = 0.3602 >= 0.0500  removing sex0male1female
p = 0.2324 >= 0.0500  removing Level of education

     Source |       SS       df       MS              Number of obs =      19
-------------+------------------------------           F(  0,    18) =    0.00
      Model |          0     0           .            Prob > F      =       .
   Residual |  767.684211    18  42.6491228           R-squared     =  0.0000
-------------+------------------------------           Adj R-squared =  0.0000
      Total |  767.684211    18  42.6491228           Root MSE      =  6.5306

------------------------------------------------------------------------------
  State Difference |      Coef.   Std. Err.      t    P>|t|     [95% Conf. Interval]
-------------+----------------------------------------------------------------
      _cons |  -.2631579   1.498229    -0.18   0.863    -3.410821    2.884505
------------------------------------------------------------------------------
```
