# Supplementary material for: Effects of auriculotherapy on anxiety and biomarkers in Primary Health Care: a clinical trial
Source: Rev Bras Enferm. 2023 Dec 4;76(6):e20220728. doi: 10.1590/0034-7167-2022-0728pt (PMC10695056; doi:10.1590/0034-7167-2022-0728pt)
Supplement: 0034-7167-reben-76-06-e20220728-suppl02 [file 0034-7167-reben-76-06-e20220728-suppl02.pdf]

Col. stats		A	B	C	D
		Trait-Pre	Trait-Post	State-Pre	State-Post
1	Number of values	19	19	19	19
2					
3	Minimum	41.00	24.00	32.00	30.00
4	25% Percentile	47.00	40.00	39.00	39.00
5	Median	50.00	43.00	43.00	42.00
6	75% Percentile	58.00	47.00	50.00	50.00
7	Maximum	64.00	59.00	56.00	56.00
8					
9	Mean	52.11	43.74	44.05	44.32
10	Std. Deviation	6.691	8.143	6.988	7.095
11	Std. Error of Mean	1.535	1.868	1.603	1.628
12					
13	Lower 95% CI of mean	48.88	39.81	40.68	40.90
14	Upper 95% CI of mean	55.33	47.66	47.42	47.74
15					
16	Coefficient of variation	12.84%	18.62%	15.86%	16.01%
17					
18	Sum	990.0	831.0	837.0	842.0
19					
20	**Shapiro-Wilk normality test**				
21	W	0.9407	0.9552	0.9553	0.9617
22	P value	0.2710	0.4826	0.4838	0.6064
23	Passed normality test (alpha=0.05)?	Yes	Yes	Yes	Yes
24	P value summary	ns	ns	ns	ns

Paired t test		
1	Table Analyzed	IDATE Traço/Estado
2		
3	Column B	Trait-Post
4	vs.	vs.
5	Column A	Trait-Pre
6		
7	**Paired t test**	
8	P value	0.0007
9	P value summary	***
10	Significantly different (P < 0.05)?	Yes
11	One- or two-tailed P value?	Two-tailed
12	t, df	t=4.089, df=18
13	Number of pairs	19
14		
15	**How big is the difference?**	
16	Mean of differences	-8.368
17	SD of differences	8.921
18	SEM of differences	2.047
19	95% confidence interval	-12.67 to -4.069
20	R squared (partial eta squared)	0.4816
21		
22	**How effective was the pairing?**	
23	Correlation coefficient (r)	0.2891
24	P value (one tailed)	0.1150
25	P value summary	ns
26	Was the pairing significantly effective?	No

Paired t test		
1	Table Analyzed	IDATE Traço/Estado
2		
3	Column D	State-Post
4	vs.	vs.
5	Column C	State-Pre
6		
7	**Paired t test**	
8	P value	0.8625
9	P value summary	ns
10	Significantly different (P < 0.05)?	No
11	One- or two-tailed P value?	Two-tailed
12	t, df	t=0.1756, df=18
13	Number of pairs	19
14		
15	**How big is the difference?**	
16	Mean of differences	0.2632
17	SD of differences	6.531
18	SEM of differences	1.498
19	95% confidence interval	-2.885 to 3.411
20	R squared (partial eta squared)	0.001711
21		
22	**How effective was the pairing?**	
23	Correlation coefficient (r)	0.5700
24	P value (one tailed)	0.0054
25	P value summary	**
26	Was the pairing significantly effective?	Yes

	Correlation	A<br>STAI-Trait<br>vs.<br>S100b
1	**Pearson r**	
2	r	-0.05716
3	95% confidence interval	-0.3701 to 0.2674
4	R squared	0.003267
5		
6	**P value**	
7	P (two-tailed)	0.7332
8	P value summary	ns
9	Significant? (alpha = 0.05)	No
10		
11	**Number of XY Pairs**	38

Correlation		A<br>STAI-Trait<br>vs.<br>BDNF
1	**Pearson r**	
2	r	-0.08745
3	95% confidence interval	-0.3961 to 0.2389
4	R squared	0.007647
5		
6	**P value**	
7	P (two-tailed)	0.6016
8	P value summary	ns
9	Significant? (alpha = 0.05)	No
10		
11	**Number of XY Pairs**	38

Correlation		A STAI-Trait vs. Enolase 2
1	**Pearson r**	
2	r	0.2220
3	95% confidence interval	-0.1051 to 0.5058
4	R squared	0.04929
5		
6	**P value**	
7	P (two-tailed)	0.1804
8	P value summary	ns
9	Significant? (alpha = 0.05)	No
10		
11	**Number of XY Pairs**	38

Correlation		A
		STAI-State vs. S100b
1	**Pearson r**	
2	r	-0.2846
3	95% confidence interval	-0.5539 to 0.03863
4	R squared	0.08098
5		
6	**P value**	
7	P (two-tailed)	0.0833
8	P value summary	ns
9	Significant? (alpha = 0.05)	No
10		
11	**Number of XY Pairs**	38

Correlation		A
		STAI-State vs. BDNF
1	**Pearson r**	
2	r	-0.1309
3	95% confidence interval	-0.4325 to 0.1970
4	R squared	0.01713
5		
6	**P value**	
7	P (two-tailed)	0.4334
8	P value summary	ns
9	Significant? (alpha = 0.05)	No
10		
11	**Number of XY Pairs**	38

Correlation		A
		STAI-State vs. Enolase 2
1	**Pearson r**	
2	r	-0.06329
3	95% confidence interval	-0.3754 to 0.2617
4	R squared	0.004005
5		
6	**P value**	
7	P (two-tailed)	0.7058
8	P value summary	ns
9	Significant? (alpha = 0.05)	No
10		
11	**Number of XY Pairs**	38
