# Supplementary material for: Effects of auriculotherapy on anxiety and biomarkers in Primary Health Care: a clinical trial
Source: Rev Bras Enferm. 2023 Dec 4;76(6):e20220728. doi: 10.1590/0034-7167-2022-0728pt (PMC10695056; doi:10.1590/0034-7167-2022-0728pt)
Supplement: 0034-7167-reben-76-06-e20220728-suppl03 [file 0034-7167-reben-76-06-e20220728-suppl03.pdf]

Paired t test		
1	Table Analyzed	NSE
2		
3	Column B	*Post-treatment*
4	vs.	vs.
5	Column A	*Pre-Treatment*
6		
7	**Paired t test**	
8	P value	0.8963
9	P value summary	ns
10	Significantly different (P < 0.05)?	No
11	One- or two-tailed P value?	Two-tailed
12	t, df	t=0.1322, df=18
13	Number of pairs	19
14		
15	**How big is the difference?**	
16	Mean of differences	-8.531
17	SD of differences	281.2
18	SEM of differences	64.51
19	95% confidence interval	-144.1 to 127.0
20	R squared (partial eta squared)	0.0009706
21		
22	**How effective was the pairing?**	
23	Correlation coefficient (r)	0.6131
24	P value (one tailed)	0.0026
25	P value summary	**
26	Was the pairing significantly effective?	Yes

	Paired t test	
1	Table Analyzed	BDNF
2		
3	Column B	Post-treatment
4	vs.	vs.
5	Column A	Pre-Treatment
6		
7	**Paired t test**	
8	P value	0.2461
9	P value summary	ns
10	Significantly different (P < 0.05)?	No
11	One- or two-tailed P value?	Two-tailed
12	t, df	t=1.199, df=18
13	Number of pairs	19
14		
15	**How big is the difference?**	
16	Mean of differences	-234.6
17	SD of differences	853.1
18	SEM of differences	195.7
19	95% confidence interval	-645.8 to 176.5
20	R squared (partial eta squared)	0.07395
21		
22	**How effective was the pairing?**	
23	Correlation coefficient (r)	-0.2200
24	P value (one tailed)	0.1827
25	P value summary	ns
26	Was the pairing significantly effective?	No

Paired t test		
1	Table Analyzed	S100B
2		
3	Column B	Post-treatment
4	vs.	vs.
5	Column A	Pre-Treatment
6		
7	**Paired t test**	
8	P value	0.0023
9	P value summary	**
10	Significantly different (P < 0.05)?	Yes
11	One- or two-tailed P value?	Two-tailed
12	t, df	t=3.546, df=18
13	Number of pairs	19
14		
15	**How big is the difference?**	
16	Mean of differences	-9.998
17	SD of differences	12.29
18	SEM of differences	2.820
19	95% confidence interval	-15.92 to -4.075
20	R squared (partial eta squared)	0.4113
21		
22	**How effective was the pairing?**	
23	Correlation coefficient (r)	0.9861
24	P value (one tailed)	<0.0001
25	P value summary	****
26	Was the pairing significantly effective?	Yes

Descriptive statistics NSE		A	B
		Pre-Treatment	Post-treatment
1	Number of values	19	19
2			
3	Mean	676.5	668.0
4	Std. Deviation	304.1	332.6
5	Std. Error of Mean	69.76	76.31
6			
7	Coefficient of variation	44.95%	49.80%

Descriptive statistics BDNF		A	B
		Pre-Treatment	Post-treatment
1	Number of values	19	19
2			
3	Mean	1964	1729
4	Std. Deviation	345.1	707.9
5	Std. Error of Mean	79.18	162.4
6			
7	Coefficient of variation	17.58%	40.94%

Descriptive statistics S100B		A	B
		Pre-Treatment	Post-treatment
1	Number of values	19	19
2			
3	Mean	64.03	54.03
4	Std. Deviation	72.18	68.53
5	Std. Error of Mean	16.56	15.72
6			
7	Coefficient of variation	112.7%	126.8%
